# Transcriptional alterations during proliferation and lignification in *Phyllostachys nigra* cells

**DOI:** 10.1038/s41598-018-29645-7

**Published:** 2018-07-27

**Authors:** Shinjiro Ogita, Taiji Nomura, Yasuo Kato, Yukiko Uehara-Yamaguchi, Komaki Inoue, Takuhiro Yoshida, Tetsuya Sakurai, Kazuo Shinozaki, Keiichi Mochida

**Affiliations:** 1 Faculty of Life and Environmental Sciences, Prefectural University of Hiroshima, 5562 Nanatuka, Shobara, Hiroshima, 727-0023 Japan; 2 Biotechnology Research Center and Department of Biotechnology, Toyama Prefectural University, 5180 Kurokawa, Imizu, Toyama, 939-0398 Japan; 3 RIKEN Center for Sustainable Resource Science, 1-7-22 Suehiro-cho, Tsurumi-ku, Yokohama, Kanagawa 230-0045 Japan; 4 Interdisciplinary Science Unit, Multidisciplinary Science Cluster, Research and Education Faculty, Kochi University, 200 Otsu, Monobe, Nankoku, Kochi, 783-8502 Japan; 5 RIKEN, Baton Zone Program, 2-1 Hirosawa, Wako, Saitama, 351-0198 Japan; 6 Kihara Institute for Biological Research, Yokohama City University, 641-12 Maioka-cho, Totsuka-ku, Yokohama, Kanagawa 244-0813 Japan; 7 Institute of Plant Science and Resources, Okayama University, Chuo 2-20-1, Kurashiki, Okayama, 710-0046 Japan

## Abstract

Highly-lignified culms of bamboo show distinctive anatomical and mechanical properties compared with the culms of other grass species. A cell culture system for *Phyllostachys nigra* has enabled investigating the alterations in cellular states associated with secondary cell wall formation during its proliferation and lignification in woody bamboos. To reveal transcriptional changes related to lignification in bamboo, we analyzed transcriptome in *P. nigra* cells treated with the synthetic auxin 2,4-dichlorophenoxyacetic acid (2,4-D) and the synthetic cytokinin benzylaminopurine (BA) by RNA-seq analysis. We found that some genes putatively involved in cell wall biogenesis and cell division were up-regulated in response to the 2,4-D treatment, and the induction of lignification by the BA treatment was correlated with up-regulation of genes involved in the shikimate pathway. We also found that genes encoding MYB transcription factors (TFs) show correlated expression patterns with those encoding cinnamyl alcohol dehydrogenase (CAD), suggesting that MYB TFs presumably regulate secondary cell wall formation in the bamboo cells. These findings suggest that cytokinin signaling may regulate lignification in *P. nigra* cells through coordinated transcriptional regulation and metabolic alterations. Our results have also produced a useful resource for better understanding of secondary cell wall formation in bamboo plants.

## Introduction

Bamboo is an ecologically and economically important grass species. It belongs to the largest subfamily, the Bambusoideae, in the grass family (Poaceae)^1,2^, which contains more than 1,500 species that are adapted to diverse climates. It has been exploited for a range of uses such as food, medicine, charcoal, and housing materials, especially in Asia^3^. Owing to their wide utility and productivity, bamboo species are increasingly regarded as a valuable resource for use in renewable energy in the development of a low-carbon society^4,5^.

It is well known that bamboo presents unique biological properties in its vegetative growth and sexual reproduction. It has a rhizome system for lateral growth and forms highly lignified woody culms for longitudinal growth without secondary growth, which are its distinguishing characteristics compared with other grass species and tree species. Moreover, bamboo species often have flowering intervals from several to more than a hundred years, which is another characteristic feature of the sexual reproduction of bamboo species. To elucidate gene regulatory networks involved in these biological phenomena observed in bamboo species, several studies have utilized transcriptome analyses, and identified spatiotemporal expressions of genes explored across different tissues and developmental stages^6–9^, which improved the understanding of the molecular mechanisms underlying the development and growth in bamboo. However, these analyses provided little information at the cellular level, and did not identify the molecular mechanisms of cellular differentiation associated with its highly-lignified culm formation.

Cell culture systems have been established in some model plant species, such as Arabidopsis T87^10^ and tobacco BY-2^11^, and exploited to investigate a wide range of aspects of plant cell biology. Recently, Ogita *et al*. established a novel xylogenic suspension culture approach in the bamboo *Phyllostachys nigra* (resource number in RIKEN BioResource Center; rpc00047) that enabled investigation of lignification in living bamboo cells^12^. The cultured *P. nigra* cells showed cell wall thickening and proliferation in response to treatment with the synthetic auxin 2,4-dichlorophenoxyacetic acid (2,4-D), and lignification occurred in response to treatment with the synthetic cytokinin benzylaminopurine (BA). After 3–5 days of induced lignification, the cells showed xylogenic differentiation, the presence of fiber-like elements with cell wall thickening, and tracheary elements with formation of perforations^12^. Elucidation of the global gene expression profiles of the suspension culture cells under lignification conditions should allow identification of the gene groups important to this process and enable the characterization of gene networks involved in lignification.

The highly conserved genic regions among *Phyllostachys* species suggest that the draft genome sequence of *P. edulis* (moso bamboo)^13^ can provide a reference genome sequence for RNA-seq-based transcriptome analyses to investigate gene expression patterns in related bamboo species whose whole genome sequences have not yet been deciphered^14^. In-depth analysis of the transcriptome dynamics in response to induced lignification in bamboo cells will provide new insights into the molecular basis of cellular differentiation.

In this study, we aimed to reveal the transcriptional regulatory networks underlying the lignification process of bamboo at the cellular level. We used RNA-seq based transcriptome analysis to obtain an overview of the gene expression of cultured *P. nigra* cells, rpc00047, and sought to identify the key pathways and transcription factors involved in its lignification process.

## Results and Discussion

### Overview of the transcriptome analysis of *P. nigra* cells

We sequenced mRNAs from control and treated *P. nigra* cells, and found that almost all of the filtered reads could be mapped to the *P. edulis* draft genome. The *P. nigra* cells were cultured with treatments of either 2,4-D or BA, and sampled at four and seven days after the initiation of the treatments. Although the cross-platform assessments suggested that Illumina and Ion Torrent would present approximately similar results in RNA-seq based transcriptome profiling, each of them could have platform-specific differentially expressed genes^15^. To minimize biases between the platforms, we applied the Illumina and Ion Torrent sequencing platforms for our RNA-seq analysis of *P. nigra* cells. From the sequenced mRNAs, we obtained 783 million reads amounting to approximately 78 gigabases in the filtered dataset; 93.22% of these sequences mapped to the *P. edulis* draft genome (Supplementary Table S1). Thus, even though we used the *P. edulis* draft genome^13^ as the reference sequence, we obtained a high rate of successful mapped reads suggesting that the *P. edulis* draft genome provides a useful reference genome sequence to analyze transcriptomes in bamboo species, probably due to their conserved genic sequences. We identified 25,443 *P. nigra* genes significantly expressed in the cells (at least one condition with average RPM values of replicated samples ≥1), which are corresponding to the counterparts annotated in the *P. edulis* draft genome. These results indicate that, in the *P. nigra* cells, genes corresponding to as much as 80% of the genes annotated in the *P. edulis* genome are detectable as significantly expressed genes (Supplementary Fig. 1a). Comparison of datasets from two duplicate samples after seven days BA treatment and sequenced on the Illumina platform gave Pearson’s correlation coefficients (PCC) of up to 0.996. Additionally, comparison of datasets from the same sample conditions using the two sequencing platforms gave high PCC values (e.g., 0.930 between control conditions); the slightly lower PCC values across sequence platforms likely reflect differences in the sequencing methodologies (Supplementary Fig. S1b). To our knowledge, this is the first study of deep transcriptome analyses of *P. nigra*, and the data from the study serve as a resource of *P. nigra* transcripts, which offer clues to identifying genes related to cellular differentiation and lignification in bamboo.

### Expression of monolignol pathway genes in response to hormonal treatment of *P. nigra* cells

**Figure 1:**
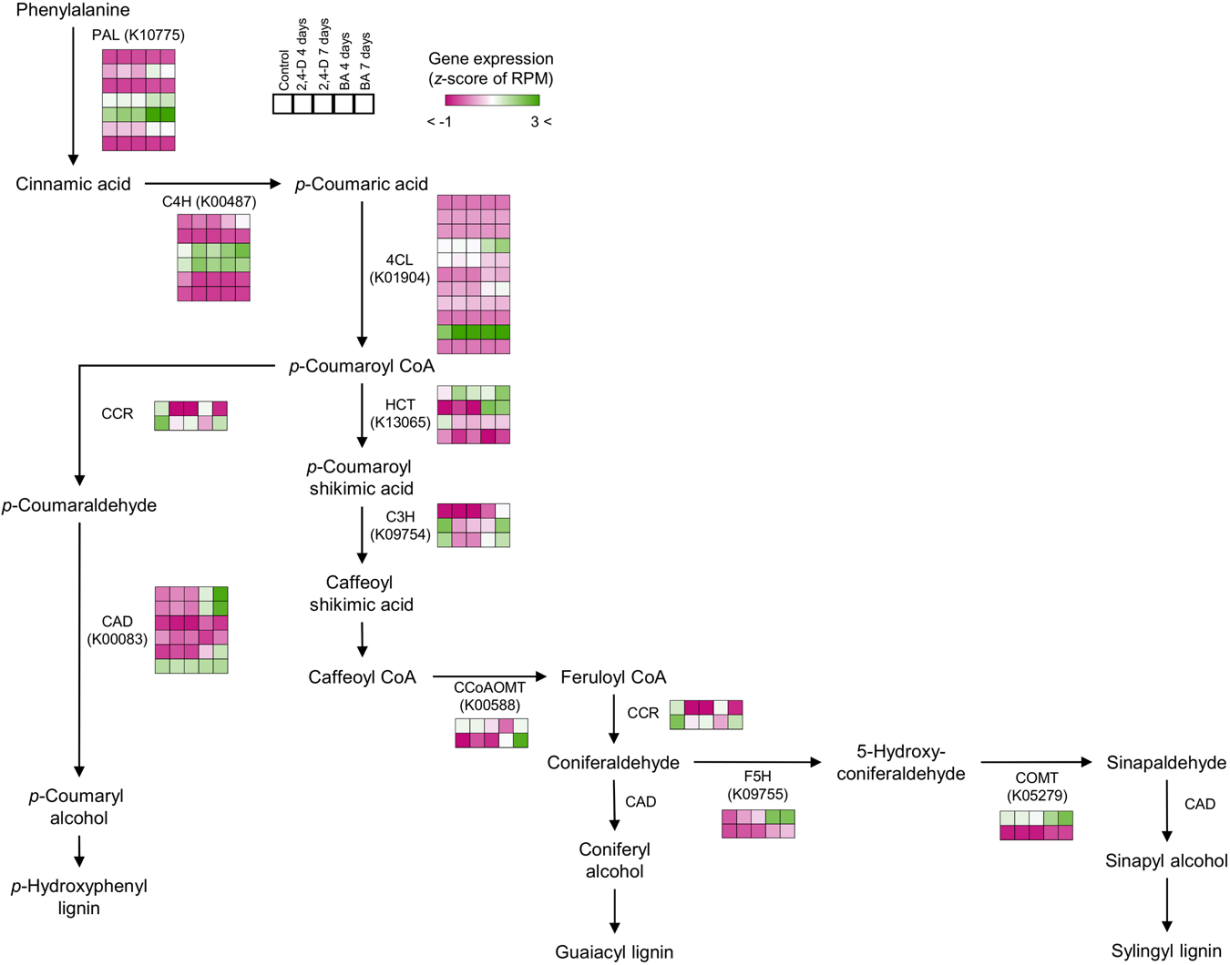
Expression of *P. nigra* genes involved in the monolignol pathway. *P. edulis* genes encoding phenylalanine ammonia lyase (PAL), cinnamate 4-hydroxylase (C4H), 4-coumarate-CoA ligase (4CL), hydroxycinnamoyl CoA:shikimate transferase (HCT), *p*-coumarate 3-hydroxylase (C3H), caffeoyl CoA *O*-methyltransferase (CCoAOMT), cinnamoyl CoA reductase (CRR), ferulate 5-hydroxylase (F5H), caffeic acid *O*-methyltransferase (COMT), and cinnamyl alcohol dehydrogenase (CAD) were represented in the monolignol pathway. The expression patterns of *P. nigra* genes corresponding to their homologs in *P. edulis* were estimated from the RPM values obtained from the cross-species mapping of *P. nigra* RNA-seq reads to the *P. edulis* genome. The color gradient represents normalized gene expression based on z-score of the RPM values.

An expression analysis of monolignol pathway genes in *P. nigra* showed expression of genes putatively involved in the lignification process in the cultured cells. In our previous observation, the *P. nigra* cells treated with auxins such as 2,4-D or picloram showed increased cell division and suppression of lignification, whereas cells treated with BA showed induced lignification^12^. Moreover, the *P. nigra* cells under the BA treatment presented increased signals of phloroglucinol-HCl, indicating induction of lignification, and found transcriptional changes in some xylogenesis-related genes including *PAL*, *C4H*, *CCoAOMT*, and *CCR* induced at day 4 of treatment with BA^12^. To reveal the transcriptional differences underlying the cellular responses against these hormonal treatments observed in the *P. nigra* cells, we assessed the expression patterns of *P. nigra* genes putatively involved in monolignol biosynthesis. We found that *P. nigra* genes encoding *CCR* and *C3H* were down-regulated in response to 2,4-D treatment, and that some downstream genes in the monolignol biosynthesis pathway, such as *CAD*, *F5H*, and *COMT*, were up-regulated in response to BA treatment (Fig. 1). Specifically, we found that three genes putatively encoding *CAD* (homologous to PH01000043G2130, PH01000043G2150, and PH01003504G0010 in *P. edulis*), *F5H* (homologous to PH01000012G2270 in *P. edulis*), and *COMT* (homologous to PH01000383G0390 in *P. edulis*) showed a clear response to BA treatment, suggesting their coordinated gene expressions associated with cellular lignification in *P. nigra* cells. We also found that some genes, such as those encoding *PAL*, *C4H*, and *4CL*, were up-regulated in response to both 2,4-D and BA treatments. These results suggest that the specific up-regulation of genes encoding *CAD*, *F5H*, and *COMT* in response to BA treatment may presumably be molecular differences associated with the differential cellular responses. For some copies in each gene group, our results are consistent with the cellular responses that initiate differentiation and lignification as well as the expression patterns of genes investigated in the previous study of *P. nigra* cells^12^. We also found some gene copies, even those encoding the same enzyme that showed different patterns of expression and/or a low level of expression in all conditions, suggesting that subfunctionalization and/or nonfunctionalization may have caused diversification of the expression patterns of these putative paralogous genes. Through our transcriptome analysis, we identified genes involved in the monolignol pathway in *P. nigra* that were expressed consistently with lignification, suggesting these genes and orthologs will be useful expression markers for monitoring the lignification process in bamboo species.

### *P. nigra* gene expression in response to 2,4-D and BA treatments

**Figure 2:**
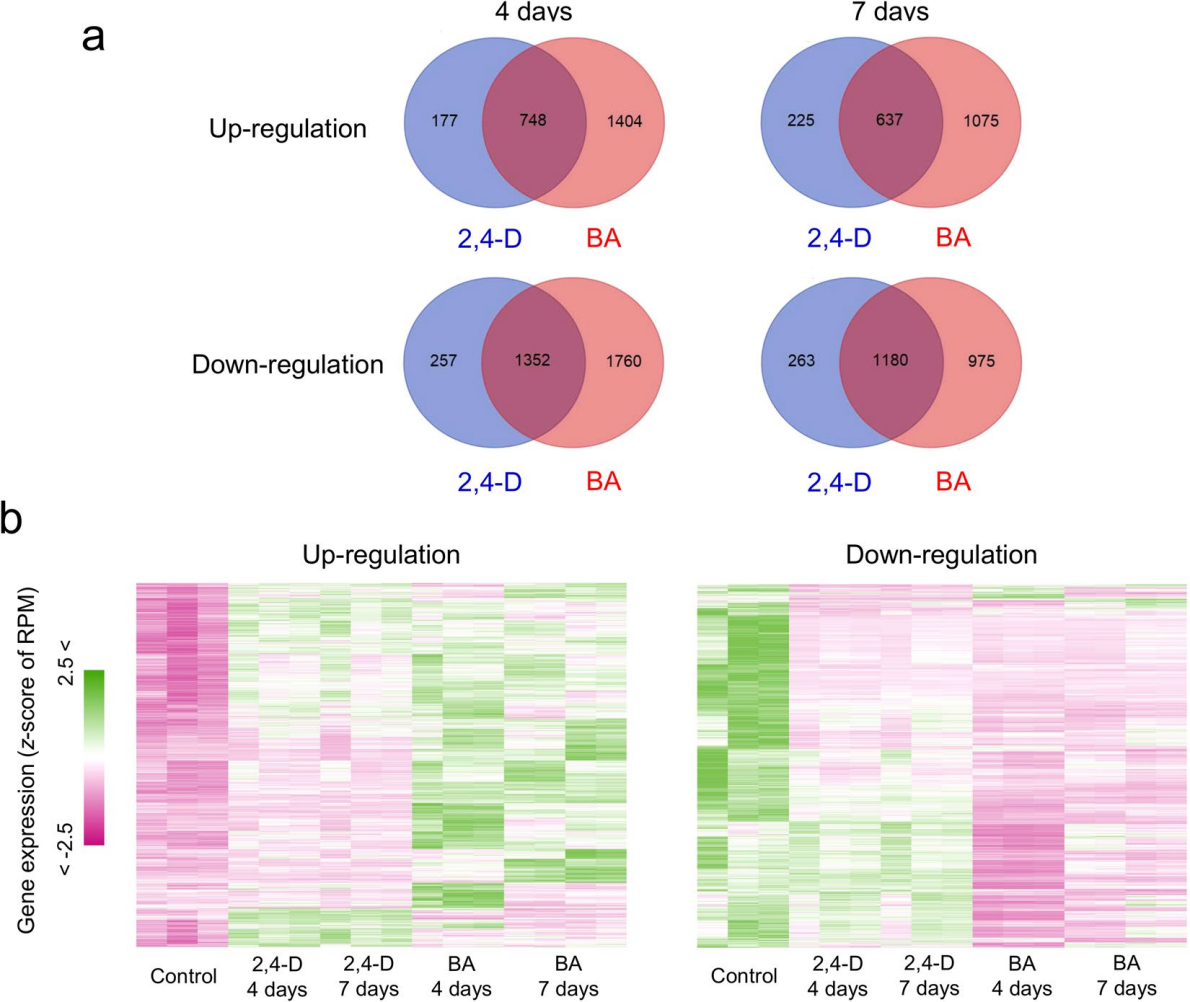
Differentially expressed genes (DEGs) in *P. nigra* cells in response to the 2,4-D and BA treatments. (**a**) Venn diagrams representing the number of up-regulated and down-regulated DEGs in each of the hormonal conditions. (**b**) Heat maps of a hierarchical clustering analysis based on the average linkage method of gene expression profiles across samples using the up-regulated and down-regulated genes. The color gradient represents normalized gene expression based on the z-score of the RPM values.

Comparing the gene expression in cells cultured under control and hormone-treated conditions, we identified a number of DEGs in the latter treatments. We first sought to identify the genes whose expression was responsive to 2,4-D and BA treatments by comparing up- and down-regulated genes. Our results showed that BA treatment triggered a change in expression of a larger number of genes than the 2,4-D treatment (Fig. 2a). Using the threshold adjusted p-value < 1e-03, 1,404 genes were found to be specifically up-regulated in samples from the 4-day treatment with BA compared with that in the control, whereas 177 genes were specifically up-regulated in 4-day treatments with 2,4-D; in addition, 748 genes were up-regulated in both treatment groups (Fig. 2a). In view of clustered gene expression patterns among DEGs, we found that more genes respond significantly to BA than to 2,4-D (Fig. 2b). These results indicate that many genes show altered expression patterns in response to the hormone treatments in *P. nigra* cells, which suggests that a broad range of cellular systems are influenced by the hormone treatments.

### Functional classification of the DEGs

**Figure 3:**
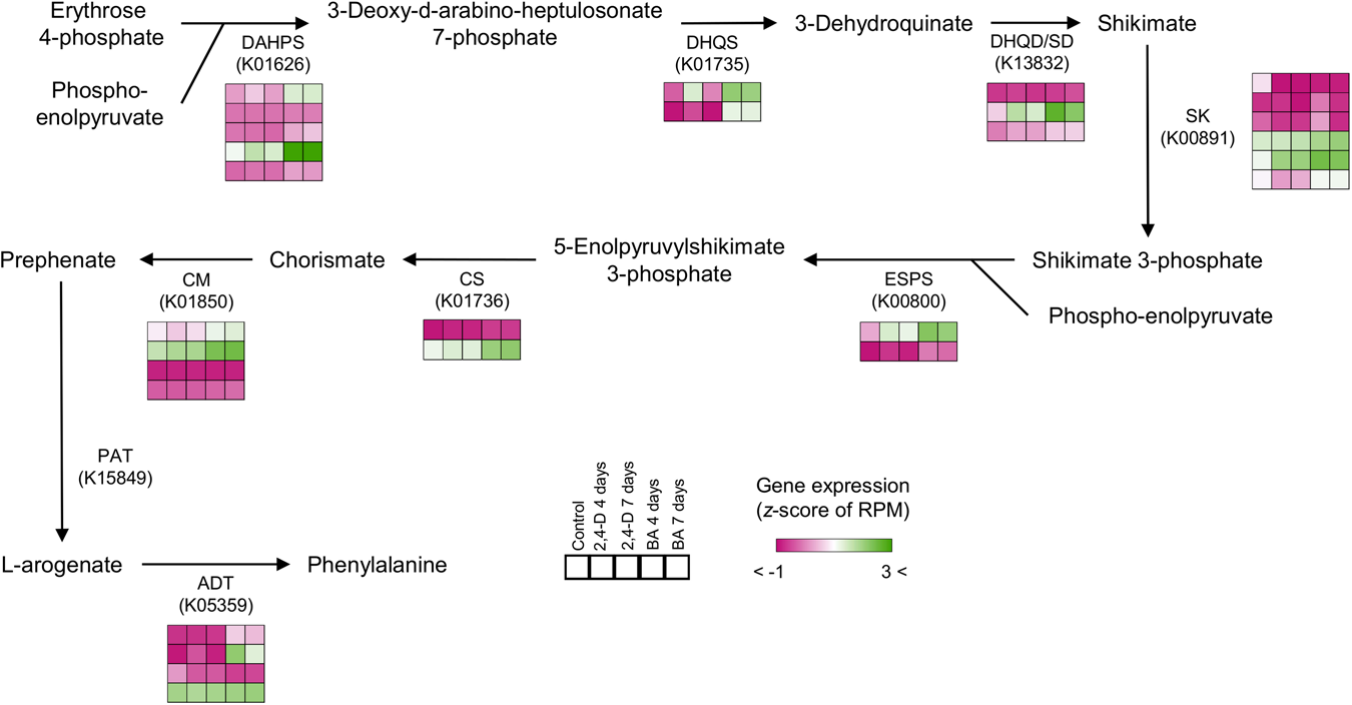
Expression of *P. nigra* genes involved in shikimate acid and phenylalanine biosynthesis. The *P. edulis* genes encoding 2-dehydro-3-deoxyphosphoheptonate aldolase (DAHPS), dehydroquinate synthase (DHQS), DHQD/SD (3-dehydroquinate dehydratase/shikimate-NADP oxidoreductase), shikimate kinase (SK), 5-enolpyruvylshikimate-3-phosphate synthase (ESPS), chorismate synthase (CS), chorismate mutase (CM), and arogenate dehydratase (ADT) were represented in the shikimate acid and phenylalanine pathways. KEGG Orthology IDs are also shown. The color gradient represents normalized gene expression based on the z-score of the RPM values.

Through enrichment analysis of functional classes and pathways among the DEGs up-regulated in response to the 2,4-D and BA treatments, we assessed the cellular functions that might be associated with the cellular differentiation induced by the hormone treatments in the *P. nigra* cultured cells. Among the DEGs specifically up-regulated in response to the 2,4-D treatment, we found that the genes encoding galactose transferase (MapMan #10.3.1.1) and cellulose synthase (MapMan #10.2.1) were enriched in both the 4-day and 7-day treatments (Table 1). We also found that genes putatively encoding cellulose synthase A (KEGG: K10999) were enriched with the 7-day treatment. These findings indicate that *P. nigra* cells treated with 2,4-D induce expression of the genes related to cell wall biogenesis, which is consistent with our previous observation of thickening and proliferation of the cells in response to the 2,4-D treatment. We also found that laccase activity (KEGG: K05909) was an enriched function among DEGs in response to the 2,4-D treatment. Because secondary wall-associated laccases are required for lignification by catalyzing oxidation of phenolic compounds^16,17^, the 2,4-D treatments may partially activate the process for secondary cell wall formation in *P. nigra* cells. Among the DEGs specifically up-regulated in response to the BA treatment, we found some enriched functions related to genes encoding enzymes involved in amino acid biosynthesis. Specifically, we found an enrichment of genes involved in the shikimate pathway^18^ for biosynthesis of aromatic amino acids (MapMan: #13.1.6.5.1, #13.1.6.5.5, and #13.1.6.1.1, KEGG: K01626), suggesting specific activation of the shikimate pathway in response to the BA treatment (Fig. 3), which can occur prior to monolignol biosynthesis (Fig. 1) and the subsequent lignification observed in the *P. nigra* cells. We also found over-representation of the genes related to transporter activities in the DEGs upregulated in response to the BA treatment (MapMan: #34.99, #34.15, and #34.16, KEGG: K03301 and K03549) (Table 2), suggesting that BA treatment activates genes encoding transporters and subsequently affects cellular logistics in the *P. nigra* cells. The list of upregulated DEGs classified to the MapMan binode with the prefix #34 (transport) has showcased genes homologous to various types of transporters, including 12 genes homologous to ATP-binding cassette (ABC) transporters (Supplementary Table S2), which may be involved in the transportation of monolignols^19^. Specifically, four genes encoding putative G family ABC transporters (homologous to PH01000231G0750, PH01002712G0070, PH01002800G0200, and PH01003385G0160 in *P. edulis*) might be involved in transporting monolignols from the cytoplasm to the cell wall for polymerization in the *P. nigra* cells. In Arabidopsis, a member of G family ABC transporter, AtABCG29, shows p-coumaryl alcohol transporter activity, and is the first monolignol transporter reported^20,21^. More recently, expression analysis of transporter encoding genes during tracheary element differentiation in cultured Arabidopsis cells suggested that four Arabidopsis ABC transporters; AtABCG11, AtABCG22, AtABCG36, and AtABCG29, may also be involved in lignification as candidate monolignol transporters^22^. The *P. nigra* cell culture system will provide a useful resource to identify ABC transporters that regulate cellular localization of monolignols in bamboo species, which may offer us novel insights into the evolution of the monolignol biosynthetic pathway in higher plants. In the DEGs upregulated in response to the BA treatment, we also found significant enrichment of a number of genes classified into an unknown functional category (MapMan: #35.2) (Table 2), suggesting that the BA treatment may affect the expression of genes involved in various cellular functions that remain unexplored. On the whole, these results illuminate the transcriptional alterations of *P. nigra* cells in response to both the 2,4-D and BA treatments, providing a comprehensive list of genes that may be involved in cellular functions related to proliferation and lignification (Supplementary Tables S3 and S4).

**Table 1:** Enriched functions found in the up-regulated genes under the 2,4-D condition in the *P. nigra* cells.

Days	Ontology	Description	P-value	Resources
4 days	10.3.1.1	cell wall.hemicellulose synthesis.xyloglucan.XXXG galactose Transferase	9.10E-05	MapMan
	27.3.63	RNA.regulation of transcription.PHD finger transcription factor	0.000107	
	30.11	signalling.light	0.001734	
	10.2.1	cell wall.cellulose synthesis.cellulose synthase	0.002123	
	35.2	not assigned.unknown	0.002507	
	K11665	DNA helicase INO80 [EC:3.6.4.12]	3.05E-05	KEGG
	K12619	5′-3′ exoribonuclease 2 [EC:3.1.13.-]	0.000181	
7 days	10.2.1	cell wall.cellulose synthesis.cellulose synthase	8.39E-07	MapMan
	27.3.63	RNA.regulation of transcription.PHD finger transcription factor	5.28E-06	
	10.3.1.1	cell wall.hemicellulose synthesis.xyloglucan.XXXG galactose Transferase	0.000147	
	11.1.1.1	lipid metabolism.FA synthesis and FA elongation.Acetyl CoA Carboxylation.homomeric Enzyme	0.000293	
	35.1.12	not assigned.no ontology.pumilio/Puf RNA-binding domain-containing protein	0.000548	
	13.1.3.1.1	amino acid metabolism.synthesis.aspartate family.asparagine.asparagine synthetase	0.000725	
	31.1.1.3.8	cell.organisation.cytoskeleton.Myosin.Class VII	0.000725	
	35.2	not assigned.unknown	0.000897	
	11.9.3.3	lipid metabolism.lipid degradation.lysophospholipases.glycerophosphodiester phosphodiesterase	0.00424	
	29.2.2.3.1	protein.synthesis.ribosome biogenesis.Pre-rRNA processing and modifications.snoRNPs	0.00424	
	K18442	brefeldin A-inhibited guanine nucleotide-exchange protein	1.87E-05	KEGG
	K10999	cellulose synthase A [EC:2.4.1.12]	2.27E-05	
	K11665	DNA helicase INO80	4.93E-05	
	K13462	guanine nucleotide-exchange factor	4.93E-05	
	K17943	pumilio RNA-binding family	0.000147	
	K12879	THO complex subunit 2	0.000147	
	K11262	acetyl-CoA carboxylase/biotin carboxylase 1 [EC:6.4.1.2, EC:6.3.4.14, EC:2.1.3.15]	0.000293	
	K01953	asparagine synthase (glutamine-hydrolysing) [EC:6.3.5.4]	0.000486	
	K01090	protein phosphatase [EC:3.1.3.16]	0.001341	
	K12617	DNA topoisomerase 2-associated protein PAT1	0.001341	
	K05909	laccase [EC:1.10.3.2]	0.003104

**Table 2:** Enriched functions found in the up-regulated genes under the BA condition in the *P. nigra* cells.

Days	Ontology	Description	P-value	Resources
4 days	35.2	not assigned.unknown	2.20E-16	MapMan
	34.99	transport.misc	2.11E-05	
	30.2.6	signalling.receptor kinases.leucine rich repeat VI	9.75E-05	
	8.1.5	TCA/org transformation.TCA.2-oxoglutarate dehydrogenase	0.000116	
	13.1.6.5.1	amino acid metabolism.synthesis.aromatic aa.tryptophan.anthranilate synthase	0.000224	
	13.1.3.4.11	amino acid metabolism.synthesis.aspartate family.methionine.S-adenosylmethionine synthetase	0.000327	
	35.1.1	not assigned.no ontology.ABC1 family protein	0.000336	
	29.2.4	protein.synthesis.elongation	0.000339	
	13.1.6.5.5	amino acid metabolism.synthesis.aromatic aa.tryptophan.tryptophan synthase	0.00039	
	K03301	ATP:ADP antiporter, AAA family	5.16E-05	KEGG
	K03327	multidrug resistance protein, MATE family	5.53E-05	
	K00799	glutathione S-transferase [EC:2.5.1.18]	0.000149	
	K00600	glycine hydroxymethyltransferase [EC:2.1.2.1]	0.000224	
	K04043	molecular chaperone DnaK	0.000224	
	K13024	inositol-hexakisphosphate/diphosphoinositol-pentakisphosphate 1-kinase [EC:2.7.4.24]	0.000224	
	K00164	2-oxoglutarate dehydrogenase E1 component [EC:1.2.4.2]	0.000327	
	K07513	acetyl-CoA acyltransferase 1 [EC:2.3.1.16]	0.000327	
	K10592	E3 ubiquitin-protein ligase HUWE1 [EC:2.3.2.26]	0.000327	
	K13034	L-3-cyanoalanine synthase/cysteine synthase [EC:2.5.1.47, EC:4.4.1.9]	0.000327	
	K14492	two-component response regulator ARR-A family	0.00039	
7 days	35.2	not assigned.unknown	2.20E-16	MapMan
	12.2.1.1	N-metabolism.ammonia metabolism.glutamate synthase.ferredoxin dependent	1.27E-06	
	13.1.6.1.1	amino acid metabolism.synthesis.aromatic aa.chorismate.3-deoxy-D-arabino-heptulosonate 7-phosphate synthase	6.17E-06	
	34.99	transport.misc	8.99E-05	
	13.1.3.4.11	amino acid metabolism.synthesis.aspartate family.methionine.S-adenosylmethionine synthetase	0.000148	
	30.2.6	signalling.receptor kinases.leucine rich repeat VI	0.000184	
	34.15	transport.potassium	0.000202	
	25.1	C1-metabolism.glycine hydroxymethyltransferase	0.000227	
	34.16	transport.ABC transporters and multidrug resistance systems	0.000319	
	K00284	glutamate synthase (ferredoxin) [EC:1.4.7.1]	1.27E-06	KEGG
	K01626	3-deoxy-7-phosphoheptulonate synthase [EC:2.5.1.54]	6.17E-06	
	K03327	multidrug resistance protein, MATE family	8.13E-06	
	K01278	dipeptidyl-peptidase 4 [EC:3.4.14.5]	3.79E-05	
	K03549	KUP system potassium uptake protein	3.88E-05	
	K00600	glycine hydroxymethyltransferase [EC:2.1.2.1]	7.97E-05	
	K13034	L-3-cyanoalanine synthase/cysteine synthase [EC:2.5.1.47, EC:4.4.1.9]	0.000148	
	K01904	4-coumarate–CoA ligase [EC:6.2.1.12]	0.000347	
	K00789	S-adenosylmethionine synthetase [EC:2.5.1.6]	0.00036	
	K01783	ribulose-phosphate 3-epimerase [EC:5.1.3.1]	0.00036	

### Changes in expression of transcription factor genes in response to BA treatment in *P. nigra* cells

We identified transcription factors (TFs) possibly involved in cellular lignification by a comparison of the expression patterns of *P. nigra* genes putatively encoding transcription factors with those of genes encoding enzymes that catalyze downstream processes in the monolignol pathways, such as CAD, F5H, and COM. This comparison yielded 1,663 genes that putatively encode DNA-binding domains (DBDs) in genes from 60 TF families that are annotated in the *P. edulis* genome. In addition, based on a comparison of *P. edulis* and Arabidopsis genomes, we identified genes homologous to Arabidopsis TFs for the promoters involved in cellulose, xylan, and lignin biosynthesis during secondary cell wall formation. These Arabidopsis genes were identified by a yeast one-hybrid assay^23^. Based on the co-expression patterns of these TFs with genes encoding enzymes involved in downstream monolignol pathways, we identified 18 genes putatively encoding TFs, including 7 MYB family genes, three ERF/AP2 family genes, two calmodulin-binding transcription activator (CAMTA), two GRAS family genes, and one bHLH family gene, which showed co-expression patterns with genes putatively encoding CAD, F5H or COMT (PCC ≥ 0.8) (Table 3). We found that the TF genes were homologous to the AtMYB85 and AtMYB20 genes of Arabidopsis. AtMYB85 is a known lignin-specific transcription factor that regulates lignin biosynthesis genes to activate secondary cell wall formation in Arabidopsis^24,25^. AtMYB20 also regulates secondary cell wall biosynthesis and is induced by NAC transcription factors that regulate secondary cell wall biosynthesis such as SND1, NSTs, and VNDs in Arabidopsis^24^. A co-expression network analysis of genes expressed during internode development in rice identified orthologs of MYB85 and MYB20 as important for secondary cell wall development^26^. These findings suggest that the transcriptional regulatory network for secondary cell wall formation in *P. nigra* cells might include some TFs conserved between dicot and monocot plants. Moreover, genes in the ERF/AP2 family are homologous to RAP2.12 in Arabidopsis, which is known to have a role in ethylene signaling^27,28^, and possibly regulates the final stages of xylogenesis through ethylene signaling^29,30^. The possible activation of genes for xylogenesis after the induction of lignification in bamboo cells suggests that secondary cell wall formation and subsequent xylogenesis might be coordinated through CK/ethylene crosstalk in bamboo cells^31,32^. We also identified one gene encoding bHLH transcription factors that showed correlated expression with genes for monolignol biosynthesis. This gene was homologous to bHLH105, which encodes IAA-LEUCINE RESISTANT3 (ILR3) that has a crucial role in Fe homeostasis through direct interaction with bHLH34 and bHLH104^33^. It has been reported that both bHLH transcription factors participate in an Arabidopsis gene regulatory network for secondary cell wall biosynthesis^23,34^.

**Table 3:** Transcription factors whose gene expression patterns correlated with the genes involved in monolignol biosynthesis in *P. nigra*.

IDs of moso bamboo homologs	Closest homologs			Promoters			Gene expression (z-score of RPM)					Correlation coefficients with expression patterns of *P. nigra* genes involved in the monolignol biosynthesis				
	Rice	Arabidopsis	Gene symbols in Arabidopsis	cellulose	lignin	xylan	Control	2,4-D 4 days	2,4-D 7 days	BA 4 days	BA 7 days	CAD	CAD	CAD	F5H	COMT
PH01000030G0050	LOC_Os04g50770	AT1G79180	MYB63	✔	✔		0.39	−1.17	−1.21	0.89	1.10	0.68	0.72	0.68	0.41	0.83
PH01000060G0800	LOC_Os09g36250	AT4G22680	MYB85	✔	✔		−0.61	−0.59	−0.54	−0.23	1.98	0.94	0.90	0.92	0.69	0.87
PH01000150G0510	LOC_Os08g39830	AT1G73730	EIL3			✔	−1.69	−0.35	0.36	1.32	0.36	0.56	0.56	0.58	0.90	0.42
PH01000210G1070	LOC_Os02g54160	AT1G53910	RAP2.12		✔		−0.69	−0.89	−0.65	0.48	1.75	0.99	0.98	0.98	0.83	0.96
PH01000348G0830	LOC_Os05g38140	AT5G54680	bHLH105	✔	✔	✔	−0.13	−1.10	−0.94	0.58	1.60	0.91	0.92	0.90	0.66	0.96
PH01000847G0490	LOC_Os09g23620	AT1G66230	MYB20		✔		−0.84	−0.77	−0.83	1.16	1.29	0.94	0.96	0.95	0.91	0.95
PH01001102G0050	LOC_Os06g09390	AT1G53910	RAP2.12		✔		−1.22	−0.25	−0.35	0.00	1.83	0.91	0.87	0.90	0.85	0.77
PH01001197G0410	LOC_Os10g22430	AT5G48150	PAT1		✔		−1.38	−0.17	−0.53	1.60	0.47	0.70	0.73	0.73	0.92	0.66
PH01001287G0090	LOC_Os04g43680	AT1G06180	MYB13		✔		−1.10	−0.12	−0.90	1.66	0.47	0.73	0.78	0.77	0.87	0.75
PH01001342G0270	LOC_Os06g14670	AT1G66230	MYB20		✔		−0.92	−0.91	−0.48	1.62	0.69	0.81	0.84	0.82	0.92	0.80
PH01001360G0240	LOC_Os03g09100	AT5G64220	CAMTA2		✔		−1.31	−0.43	−0.10	0.08	1.76	0.90	0.86	0.89	0.87	0.74
PH01001360G0260	LOC_Os03g09100	AT5G64220	CAMTA2		✔		−1.61	−0.11	0.22	−0.04	1.54	0.76	0.72	0.76	0.82	0.57
PH01001873G0040	LOC_Os12g39220	AT1G24625	ZFP7		✔	✔	−0.84	−0.68	−0.44	1.91	0.05	0.61	0.66	0.64	0.84	0.63
PH01002680G0080	LOC_Os06g14670	AT1G66230	MYB20		✔		−0.73	−1.09	−0.59	1.35	1.05	0.87	0.89	0.87	0.87	0.88
PH01003093G0130	LOC_Os09g36250	AT4G22680	MYB85	✔	✔		−0.47	−0.72	−0.84	0.15	1.88	0.97	0.96	0.96	0.71	0.97
PH01003592G0180	LOC_Os03g47140	AT2G22840	GRF1		✔	✔	−1.75	0.71	−0.47	0.59	0.92	0.64	0.64	0.67	0.81	0.52
PH01003923G0110	LOC_Os01g12440	AT1G50640	ERF3	✔		✔	−1.35	−0.58	−0.29	1.49	0.73	0.78	0.79	0.80	0.98	0.70
PH01004866G0030	LOC_Os10g22430	AT5G48150	PAT1		✔		−1.86	0.20	−0.06	0.89	0.83	0.67	0.67	0.70	0.91	0.53

^*^Interactions of Arabidopsis TFs for promoters involved in cellulose, xylan, and lignin biogenesis summarized in Kumar *et al*.^34^ based on the Y1H data from Taylor-Teeples *et al*.^23^.

### Metabolic differences of *P. nigra* cells treated with 2,4-D and BA

**Figure 4:**
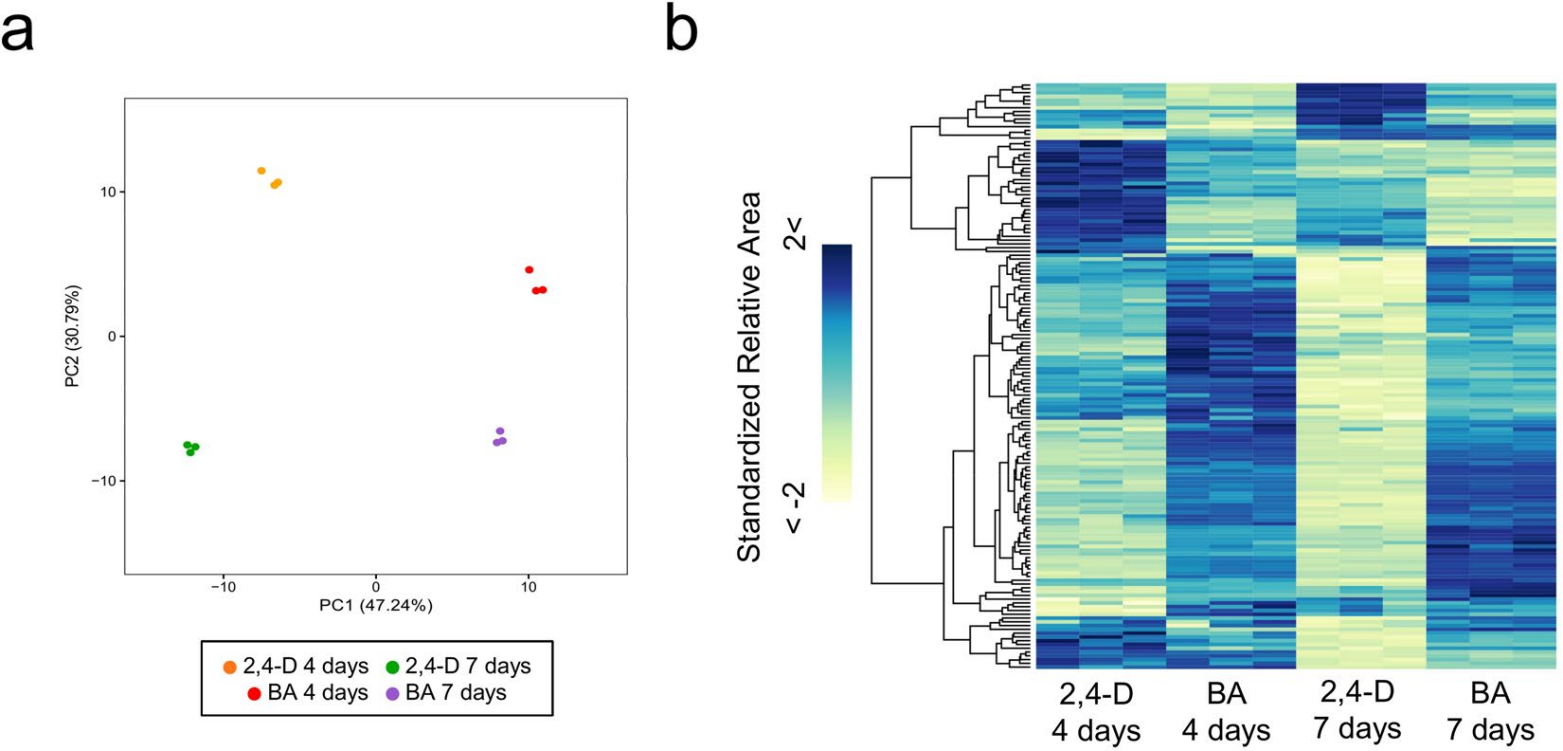
Metabolomic differences in *P. nigra* cells treated with 2,4-D and BA. (**a**) PCA of the metabolomic data of the *P. nigra* cells from the 4-day and 7-day treatments with 2,4-D and BA. (**b**) Hierarchically clustered heat map representation of 218 metabolites (lines) across the 4 conditions.

**Figure 5:**
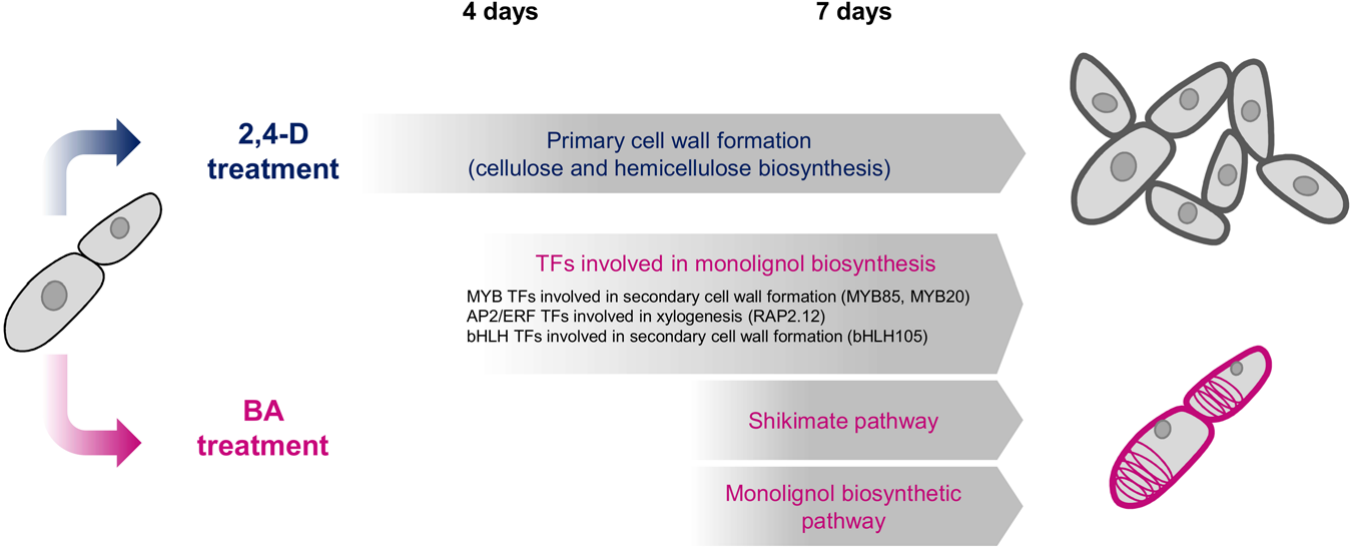
Summary of cellular and transcriptomic alterations in *P. nigra* cells in response to the 2,4-D and BA treatments.

In the xylogenic suspension culture, *P. nigra* cells present differential metabolomic properties during proliferation and lignification in response to treatment with 2,4-D and BA. To reveal metabolic changes and explore its relationship with the transcriptional changes occurring during cellular differentiation, we performed a widely targeted metabolome analysis using CE-MS, with samples of *P. nigra* cells from the 4-day and 7-day treatments with 2,4-D and BA, respectively, and obtained a metabolome profile dataset composed of accumulation patterns of 214 compounds (Supplementary Table S5). In the widely targeted metabolome dataset, we found that amino acids synthesized through the shikimate pathway, such as phenylalanine (C_0075: Phe in Supplementary Table S6), tryptophan (C_0102: Trp in Supplementary Table S6), and tyrosine (C_0088: Tyr in Supplementary Table S6), are significantly increased their relative size of MS peaks under the BA conditions (p-value of Welch’s t-test < 0.001), suggesting that BA activates the shikimate pathway (Fig. 3), and consequently increases phenylalanine and tyrosine for monolignol biosynthesis in the *P. nigra* cells (Fig. 2). Moreover, we observed clear metabolic differences between the 4 sample conditions (Fig. 4a,b), suggesting significant metabolic alteration during the cellular differentiation process. Comparing the metabolome profiles of the *P. nigra* cells treated with 2,4-D and BA, we identified 120 and 131 metabolites that were differentially accumulated in the cells from the 4-day and 7-day treatments (p-value of Welch’s t-test <0.05), respectively, and found that metabolites of amino acids, nucleotide, sugars, and lipids were abundantly accumulated in the cells treated with BA (Supplementary Table S6). Based on the findings of our transcriptome analysis as well as widely-targeted metabolome analysis of the *P. nigra* cells treated with 2,4-D and BA, we obtained a comprehensive view of the transcriptomic and metabolic alterations occurring in response to the hormonal treatments, which induce proliferation and lignification in a bamboo species (Fig. 5). In response to the 2,4-D treatment, the *P. nigra* cells activate genes related to cell division and cell growth to promote their proliferation. During this process, they also activate genes associated with biosynthesis of cellulose and hemicellulose, which promote cell wall thickening through primary cell wall formation. In contrast, with BA treatment, *P. nigra* cells activate genes encoding TFs associated with secondary cell wall formation and the shikimate pathway to synthesize aromatic amino acids, followed by monolignol pathway genes to synthesize monolignol precursors.

## Conclusions

Our transcriptome analysis of cultured *P. nigra* cells that had been induced to undergo proliferation and lignification, identified changes to transcriptional regulatory networks and cellular metabolism, which were presumably related. Functional analyses of the genes encoding TFs that might be involved in lignification in *P. nigra* will undoubtedly identify regulatory factors for lignification in bamboos. Comprehensive investigation of the lignification process using the *P. nigra* cell culture system in combination with various -omics analyses will provide a valuable framework for accelerating our understanding of the cellular systems regulating lignification in bamboo species.

## Materials and Methods

### Cell culture

Bamboo (*P. nigra*) cells were maintained in suspension culture in modified half-strength Murashige and Skoog (MS) liquid medium^35^ supplemented with 3 μM 2,4-D, as described previously^36^. Subcultures were established in 100 ml liquid medium in a 300-ml flask and maintained on a rotary shaker (110 rpm) in the dark at 25 °C. To maintain stable morphology and synchronous growth of the cells, the sedimented cell volume was adjusted to 2.5% every two weeks as described previously^37^.

To promote lignification in the cells, 2-week-old cell cultures were transferred to half-strength MS medium supplemented with 10 μM benzylaminopurine (BA) and 3% (w/v) sucrose (lignification conditions) and cultured as described above^12^.

### Sample preparation for metabolome analysis

The *P. nigra* cells were immediately frozen in liquid nitrogen and stored at −80 °C until metabolite extraction. Cell samples were weighed and homogenized by Shake Master, BMS-M10N21 (BioMedicalScience, Japan) three times at 1,500 rpm for 2 min, after addition of 500 μl of ice-cold methanol containing 50 μM methionine sulfone as an internal standard. The homogenates were mixed with 500 μl of chloroform and 200 μl of ice-cold Milli-Q water. After centrifugation at 2,300 × g for 5 min at 4 °C, the supernatant was centrifugally filtrated with a Millipore Ultrafree-MC PLHCC HMT Centrifugal Filter Device, 5 kDa (Millipore, Billerica, MA, USA). The filtrate was dried and dissolved in 50 μl of Milli-Q water, and analyzed by CE-TOFMS.

### RNA extraction

Total RNA was extracted from *P. nigra* cells using NucleoSpin RNA (Macherey-Nagel, USA), and quality was checked using an Agilent 2100 Bioanalyzer (Agilent, USA).

### Library preparation and sequencing

For Illumina based RNA-sequencing, sequencing libraries were constructed using a TruSeq Sample Preparation Kit (Illumina, Inc.) according to the manufacturer’s instructions. The sequencing libraries were sequenced using a Hiseq2000 sequencer by the paired-end sequencing method for sequences 100 bp in length. For ion torrent based RNA-sequencing, poly(A) + RNAs were purified using the MicroPoly(A)Purist™ Kit (Life Technologies, USA) according to the manufacturer’s instructions. Sequencing libraries were obtained using the Ion Total RNA-Seq Kit v2 (Life Technologies, USA) according to the manufacturer’s instructions with Ion Xpress RNA-Seq Barcode 1–16 Kit (Life Technologies, USA). The sequencing libraries were sequenced using an Ion Proton sequencer by Ion P1 Template OT2 200 Kit v3 (Life Technologies, USA) and Ion P1 Sequencing 200 Kit v3 (Life Technologies, USA).

### Read processing

The reads from the Ion Torrent-based sequencing that passed the quality control process of the Ion Torrent system were processed by cutadapt^38^ to remove sequencing adaptors. The raw reads from the Illumina-based sequencing were trimmed and filtered based on quantity using the FASTX-Toolkit (http://hannonlab.cshl.edu/fastx_toolkit/index.html) with parameter settings of –q 30 –p 80 –v –Q 33.

### Reference genome data

The sequence dataset of the draft genome, the coding sequence (CDS), and protein sequences of *P. edulis* (*P. heterocycla* var. *pubescens*) (v1.0) were retrieved from the BambooGDB web site (http://www.bamboogdb.org/)^13,39^. To generate a dataset of structural gene annotations for the *P. edulis* genome, we mapped the CDS dataset to the draft genome using the GMAP program with default parameter settings, and estimated exon-intron coordinates for the *P. edulis* genome. A GFF file of the exon-intron coordinates was used to count reads mapped to each gene using featureCount.

### Read Mapping

The Illumina reads were mapped to the *P. edulis* genome sequence using HISAT2^40^ (version 2.0.5) with default parameter settings. The Ion Torrent reads were mapped to the *P. edulis* genome sequence using the TMAP program (Life Technologies, USA) (version 3.4.1) with parameter settings of mapall -z -o 2 stage1 map4.

### Quantification of gene expression

The featureCounts program (http://bioinf.wehi.edu.au/featureCounts/) was used to compute read counts for each gene annotated in the *P. edulis* genome and, based on the read counts, the RPM values were calculated.

### Identification of differentially expressed genes

Genes showing RPM values ≥1 in at least one sample were defined as expressed genes. Differentially expressed genes (DEGs) were calculated using the DESeq2 program^41^ running in the R package, with a threshold of adjusted p < 1 × 10^−3^.

### Functional annotation and enrichment test

To predict the functions of *P. edulis* genes, homology searches were performed using BLASTP (−e = 1e-5, −F = F) against entries of a known protein database (NCBI nr, ftp://ftp.ncbi.nih.gov/blast/db), TIGR Rice Genome Annotation Project (http://rice.plantbiology.msu.edu/), and the protein data present in TAIR release 10 (https://www.arabidopsis.org/). The KAAS web server (http://www.genome.jp/tools/kaas/)^42^ was used to map the protein sequences of *P. edulis* to metabolic pathways in the KEGG database. In the KEGG pathway mapping, BLAST was used as a search program and “hsa, dme, cel, ath, sce, cho, eco, nme, hpy, rpr, bsu, lla, cac, mge, mtu, ctr, bbu, syn, bth, dra, aae, mja, ape, osa, gmx, and vvi” as search organisms with the single directional best hit method. The Mercator pipeline (http://mapman.gabipd.org/web/guest)^43^ in the MapMan web service was used for functional classification of the protein sequences of *P. edulis* based on MapMan ontology^44^. The genes putatively encoding transcription factors in *P. edulis* were annotated based on protein-specific DNA binding domains using Hidden Markov models for 60 transcription factors in plants with an HMMER search^45–49^. Gene set enrichment analysis (GSEA) of DEGs for MapMan ontology and KEGG pathways was performed by Fisher’s exact test.

### CE-TOFMS analysis and data processing

CE-TOFMS analysis was performed using an Agilent CE system combined with a TOFMS (Agilent Technologies, Palo Alto, CA, USA) at Human Metabolome Technologies Inc. (HMT, Japan). The samples were diluted two and five times for cation and anion analysis, respectively. Cationic metabolites were separated through a fused silica capillary (50 μm internal diameter × 80 cm length) with Cation Buffer Solution, H3301-1001 (HMT, Japan). Samples were injected at a pressure of 50 mbar for 10 s with the voltage for the CE set at 27 kV. Electrospray ionization-mass spectrometry (ESI-MS) was conducted in positive-ion mode with voltage set at 3 kV. Anionic metabolites were measured through the fused silica capillary (50 μm internal diameter × 80 cm length) with Anion Buffer Solution, H3302-1021 (HMT, Japan). Samples were injected at a pressure of 50 mbar for 25 s with the voltage for the CE set at 30 kV. The ESI-MS was conducted in the negative-ion mode with the voltage set at 3.5 kV. Mass data for the cationic and anionic metabolites were acquired in a range of 50–1,000 m/z. The data were preprocessed using MasterHands software (HMT, Japan). Each metabolite was identified based on m/z and migration time of the MS peak through database search against the HMT database, and was quantified based on the peak area. Differentially accumulated metabolites were identified with a threshold of p < 0.05 in Welch’s t-test across the sample conditions.

### Data Availability

RNA-seq dataset: DDBJ Sequence Read Archive accession number DRA006159.

## Electronic supplementary material

Supplementary information

Supplementary Dataset
