# Supplementary material for: Transcriptional alterations during proliferation and lignification in Phyllostachys nigra cells
Source: Sci Rep. 2018 Jul 27;8:11347. doi: 10.1038/s41598-018-29645-7 (PMC6063902; doi:10.1038/s41598-018-29645-7)
Supplement: Supplementary file 1 — Supplementary information [file 41598_2018_29645_MOESM1_ESM.docx]

# **Supplementary Information**

# **Transcriptional alterations during proliferation and lignification in *Phyllostachys nigra* cells**

Shinjiro Ogita^1,2*^, Taiji Nomura^2^, Yasuo Kato^2^, Yukiko Uehara-Yamaguchi^3^, Komaki Inoue^3^, Takuhiro Yoshida^3^, Tetsuya Sakurai^3,4^, Kazuo Shinozaki^3^, Keiichi Mochida^3,5,6,7*^

^1^Faculty of Life and Environmental Sciences, Prefectural University of Hiroshima, 5562 Nanatuka, Shobara, Hiroshima 727-0023, Japan

^2^Biotechnology Research Center and Department of Biotechnology, Toyama Prefectural University, 5180 Kurokawa, Imizu, Toyama 939-0398, Japan

^3^RIKEN Center for Sustainable Resource Science, 1-7-22 Suehiro-cho, Tsurumi-ku, Yokohama, Kanagawa 230-0045, Japan

^4^Research and Education Faculty, Multidisciplinary Science Cluster, Interdisciplinary Science Unit, Kochi University, 200 Otsu, Monobe, Nankoku, Kochi, 783-8502 Japan

^5^RIKEN Cluster for Science, Technology and Innovation Hub, Baton Zone Program, 2-1 Hirosawa, Wako, Saitama, 351-0198, Japan

^6^Kihara Institute for Biological Research, Yokohama City University, 641-12 Maioka-cho, Totsuka-ku, Yokohama, Kanagawa 244-0813, Japan

^7^Institute of Plant Science and Resources, Okayama University, Chuo 2-20-1, Kurashiki, Okayama 710-0046, Japan

*Correspondence and requests for materials should be addressed to S.O (ogita@pu-hiroshima.ac.jp) or K.M. (keiichi.mochida@riken.jp)

**Supplementary Table S1.** Summary statistics of the RNA-seq analysis in *P. nigra* cultured cells.

**Supplementary Table S2.** Expression and functional annotation of genes upregulated in response to the BA treatments, which are assigned to transport function in the MapMan ontology.

**Supplementary Table S3.** Expression and functional annotations of the DEGs up-regulated in response to the 2,4-D treatments.

**Supplementary Table S4.** Expression and functional annotations of the DEGs up-regulated in response to the BA treatments.

**Supplementary Table S5.** Metabolite profile in the *P. nigra* cells from the 4-day and 7-day treatments with 2,4-D and BA.

**Supplementary Table S6.** Differentially accumulated metabolites in the *P. nigra* cells from the 4-day and 7-day treatments with 2,4-D and BA.


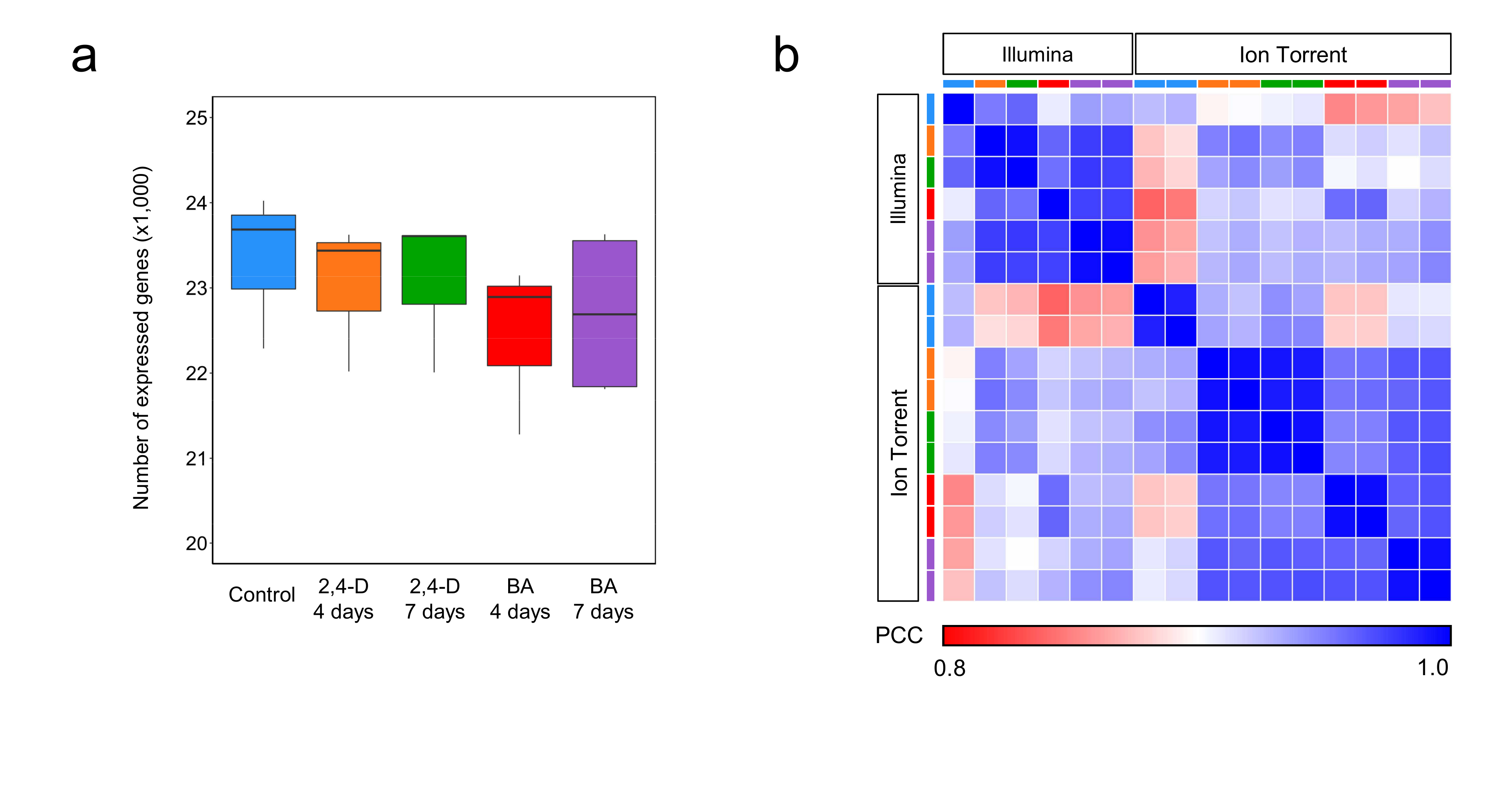


**Supplementary Fig. 1. Overview of the transcriptome of *P. nigra* cells treated with 2,4-D or BA.** (**a**) Number of expressed genes in each sample. (**b**) Correlation heatmap of gene expression profiles of the transcriptome datasets obtained from Illumina- and Ion torrent-based sequencing. Color gradient represents Pearson’s correlation coefficients between 0.8 and 1.
